# *De novo* Sequencing, Assembly and Characterization of Antennal Transcriptome of *Anomala corpulenta* Motschulsky (Coleoptera: Rutelidae)

**DOI:** 10.1371/journal.pone.0114238

**Published:** 2014-12-02

**Authors:** Haoliang Chen, Lulu Lin, Minghui Xie, Guangling Zhang, Weihua Su

**Affiliations:** Institute of Plant Protection and Agro-Products Safety, Anhui Academy of Agricultural Sciences, Hefei, China; Harvard Medical School, United States of America

## Abstract

**Background:**

*Anomala corpulenta* is an important insect pest and can cause enormous economic losses in agriculture, horticulture and forestry. It is widely distributed in China, and both larvae and adults can cause serious damage. It is difficult to control this pest because the larvae live underground. Any new control strategy should exploit alternatives to heavily and frequently used chemical insecticides. However, little genetic research has been carried out on *A. corpulenta* due to the lack of genomic resources. Genomic resources could be produced by next generation sequencing technologies with low cost and in a short time. In this study, we performed *de novo* sequencing, assembly and characterization of the antennal transcriptome of *A. corpulenta*.

**Results:**

Illumina sequencing technology was used to sequence the antennal transcriptome of *A. corpulenta*. Approximately 76.7 million total raw reads and about 68.9 million total clean reads were obtained, and then 35,656 unigenes were assembled. Of these unigenes, 21,463 of them could be annotated in the NCBI nr database, and, among the annotated unigenes, 11,154 and 6,625 unigenes could be assigned to GO and COG, respectively. Additionally, 16,350 unigenes could be annotated in the Swiss-Prot database, and 14,499 unigenes could map onto 258 pathways in the KEGG Pathway database. We also found 24 unigenes related to OBPs, 6 to CSPs, and in total 167 unigenes related to chemodetection. We analyzed 4 OBPs and 3CSPs sequences and their RT-qPCR results agreed well with their FPKM values.

**Conclusion:**

We produced the first large-scale antennal transcriptome of *A. corpulenta*, which is a species that has little genomic information in public databases. The identified chemodetection unigenes can promote the molecular mechanistic study of behavior in *A. corpulenta*. These findings provide a general sequence resource for molecular genetics research on *A. corpulenta*.

## Introduction

Chafers are important insect pests and can cause enormous economic losses in agriculture, horticulture and forestry [1]. *Anomala corpulenta* Motschulsky (Coleoptera: Rutelidae) is widely distributed in China. The larva is mainly a root-damaging pest, and it can cause plants to die when the damage is serious, while the adults feed on the leaves of such plants as apple (*Malus sieversii*), pear (*Pyrus sorotina*), Chinese ash (*Pterocarya stenoptera*), willow (*Salix babylonica*), plane tree (*Platanus orientalis*) etc. The leaves can be heavily damaged by the adults which usually emerge over a short time. Sometimes the leaves can be totally consumed, and, in more serious infestations, the branches also can be damaged [2], [3], [4]. *A. corpulenta* can cause enormous economic losses, but it is difficult to control this pest because the larvae live underground. Application of heavily and frequently used chemical insecticides (e.g., organophosphates, carbamates, neonicotinoids) is the most prevalent management method used for control of the larvae of *A. corpulenta*. This had led to an excessive use of insecticides and resulting environmental pollution [5]. Any alternative control strategy should exploit methods besides killing larvae underground using chemical insecticides. Trapping adults to reduce the larval population is considered as one alternative control method.

Chemodetection plays a key role in insect behavior, such as locating food and mates [6]. In insects, most chemosensillums are located on the antennae, and odorant binding proteins (OBPs) and chemosensory proteins (CSPs) are two main protein groups related to insect olfaction [7], [8], [9], [10], [11], [12]. Determination of the genetic pathways and specific genes involved in the pathway of detection of odorants could be beneficial for control *A. corpulenta*. However, limited genomic information is available to address these issues in the *Anomala* genus. There have been a limited number of nucleotide and protein sequences published: 377 nucleotides from 75 species and 249 proteins from 66 species have been deposited in the NCBI database. But, there are only 3 nucleotide sequences and 4 protein sequences from *A. corpulenta* that have been deposited in the NCBI database.

The development of next generation sequencing technologies (NGS) has dramatically improved the efficiency and speed of gene discovery in the past several years. All the platforms, including Illumina Solexa, Roche 454, and ABI SOLiD, can provide genomic and transcriptomic data cheaply and rapidly [13], [14], [15]. Considering the advantage of NGS, it has been used in many research areas, such as resequencing, gene discovery, small RNA expression, DNA methylation, and *de novo* transcriptome (RNA-Seq) of non-model organisms [16], [17], [18], [19], [20], [21], [22], [23], [24], [25]. Recently, the Illumina platform has been used efficiently for *de novo* transcriptome assemblies of insects such as *Bemisia tabaci* [21], *Anopheles funestus* [20], and *Locusta migratoria* [19]. These studies confirmed that Illumina deep sequencing technology can be effectively used for gene discovery and rapidly broadening our understanding of the complexity of gene regulation and gene networks.

In this study, in order to establish the antennal transcriptome of *A. corpulenta*, we performed *de novo* transcriptome sequencing on the Illumina next-generation sequencing (NGS) platform. The unigenes obtained were then annotated by BLASTing against public databases. Thereafter, the putative function of the unigenes was categorized by Gene Ontology (GO) and grouped into pathways using the Kyoto Encyclopedia of Genes and Genomes (KEGG). Some of the unigenes annotated as OBPs or CSPs were confirmed by RT-PCR.

## Materials and Methods

### Insects, RNA isolation and cDNA library preparation

Adults of *Anomala corpulenta* used in this study were obtained in May 2013 from Hefei, Anhui province, China (117.10E, 31.66N), and then about 150 pairs of antennas were collected from these adults and used for RNA isolation. Total RNA was isolated following the manufacturer’s protocol of total RNA isolation system, and then deoxyribonuclease was used to remove possible residual genomic DNA. A 2100 Bioanalyzer was used to confirm RNA integrity with a value of 9.1. Transcriptome samples were prepared following the manufacturer’s protocols. Briefly, 6 ug of total RNA were used for mRNA purified by oligo (dT) magnetic beads, and then divalent cations were used to fragment the purified mRNA into small pieces under elevated temperature in a Thermomixer at 95°C. These mRNA fragments were used for synthesis of the first strand cDNA with reverse transcriptase and random primers. DNA polymerase I and RNaseH were followed to synthesize the second strand cDNA. The synthesized cDNA fragment ends were repaired, and sequencing adapters were ligated to the cDNA fragments. Around 200 bp of these products were used to create the cDNA library by PCR.

### Deep-sequencing and *de novo* assembly

The cDNA library was deep sequencing for 4 gigabytes data. The cDNA size of the library was about 200 bp, and we sequenced both ends. Adaptor sequences, empty reads, reads containing more than 5% unknown nucleotides and low quality sequences (reads containing more than 50% bases with the Q - value ≤ 20) were removed from the raw reads, and then the clean reads were obtained. *De novo* assembly was performed by the Trinity software to generate unigenes [26]. In this study, K-mer was set at 25 bp. The raw data from Illumina deep-sequencing were deposited in the NCBI Short Read Archive (SRA) database with accession number: SRP044773.

### Sequence clustering and functional categorization of unigenes

Assembled unigenes were blast searched and annotated against nr, Swiss-Prot and KEGG databases (significant thresholds of E-value ≤ 1.0e-5). Domain-based alignments were carried out against the Cluster of Orthologous Groups (COG) database (http://www.ncbi.nlm.nih.gov/COG/) at NCBI with a cut-off E-value of ≤ 1.0e-5. Blast2go software was used to analyze Ontology term annotation (GO; http://www.geneontology.org) [27], and unigenes with annotation were distributed into three ontologies: molecular function, cellular component and biological process [28]. After obtaining GO annotation for each unigene, WEGO software was used for GO functional classification, which was to understand the distribution of gene functions of the species from the macro level [29]. Blastall software was used for COG and KEGG pathway annotation against COG and KEGG databases, respectively.

### Fragments per kb per million fragments (FPKM) value calculation and RT-qPCR

Unigene expression was calculated by the method of fragments per kb per million fragments (FPKM) [30]. The formula is:
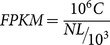
C is the number of fragments that uniquely aligned to the calculated unigene, N is the total number of fragments that uniquely aligned to all unigenes, and L is the base number of the calculated unigene.

The expression of odorant binding protein genes and chemosensory protein genes in *A. corpulenta* antennae were detected. Glyceraldehyde-3-phosphate dehydrogenase (GAPDH) was chosen as the reference gene because this gene had the most stable expression in different tissues in *A. corpulenta* (data not shown). One microgram of total RNA which had genomic DNA removed was used for synthesis of the cDNA. Beacon Designer 7.0 was used to design primers for RT-qPCR, and the Real-time PCR amplification efficiency of one cycle was calculated [31]. Before quantitative PCR, Ct values of GAPDH in each cDNA sample were tested, and then the concentrations of cDNA were adjusted according to Ct-based fold. Four biological samples were used to test the relative expression of OBPs and CSPs, and each biological sample had two technical replicates. The primers used for RT-qPCR are shown in Table S1 in Data S1. Sequences of OBPs and CSPs were deposited in Genbank with accession numbers from KM258398 to KM258404, and accession number for the reference gene GAPDH is KM267029.

### Sequence confirmation and analysis of deduced amino acid sequences of OBPs and CSPs

Confirming primers for OBPs, CSPs and other reference genes were listed in Table S1 in Data S1. RNA was first extracted from antennae of *A. corpulenta* [32], and 5 micrograms of RNA were used for synthesis of the cDNA. PCR amplifications were hot-started at 94°C for 3 min, followed by 35 cycles at 94°C for 30 s, 58°C for 30 s, 72°C for 1 min and final extension at 72°C for 10 min. PCR products were introduced into gel electrophoresis, and predicted sizes of DNA fragments were purified by Agarose Gel Extraction kit and then subcloned into vector according to the manufacturer’s protocol. The sequences of OBPs and CSPs were determined by using a 3730 DNA Analyzer. After the OBPs and CSPs were confirmed by sequencing, the nucleotide sequences were deduced to amino acid sequences, and phylogenetic trees were constructed using BEAST 2 (Bayesian evolutionary analysis sampling trees) [33] based on the OBPs and CSPs amino acid sequences in *A. corpulenta* and on all Coleoptera in the NCBI database, respectively. The protein traits of OBPs and CSPs amino acid sequences’ analysis tools were obtained from the ExPASy Proteomics website (http://expasy.org/).

## Results and Discussion

### Sequencing and Assembly

**Figure 1:**
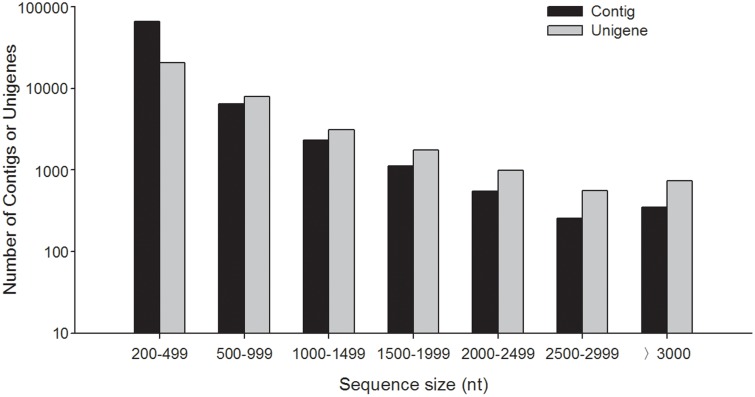
The length distribution of contigs and unigenes.

Approximate 76.7 million total raw reads were obtained by the Illumina sequencing platform and 68.9 million total clean reads were obtained after removing the adaptor and quality filtering. Those total clean reads included about 6.2 billion total clean nucleotides with a GC percentage of 38.05%. Phred quality score of ≤ Q20 level (error probability of 0.01) for the clean read was 97.49%. The clean reads were then input into trinity software, and 76,997 contigs with a mean length of 320 bp and an N50 of 524 bp (i.e., 50% of the total assembled sequence was contained in contigs of this length or longer) were produced. Contigs were then assembled into 35,656 unigenes with a mean length of 712 bp and an N50 of 1,097 bp (Table S2 in Data S1). The number of contigs and unigenes decreased with increasing size of contigs and unigenes. When the sequence size was between 200–500 bp, the number of contigs was more than the unigenes; otherwise, the number of unigenes was more than the contigs (Figure 1). Gene family clust found 8,805 clusters and 26,851 singletons in 35,656 unigenes. RT-PCR amplification was used to demonstrate the accuracy of assembled unigenes. We chose 14 unigenes to design primers, and all pairs of primers produced an expected band after RT-PCR and gel electrophoresis (primers in Table S1 in Data S1). Four OBPs and three CSPs in 14 unigenes were chosen for sequencing by Sanger sequencing, and all of them were matched with unigene sequences.

### Functional annotation of the whole transcriptome

**Figure 2:**
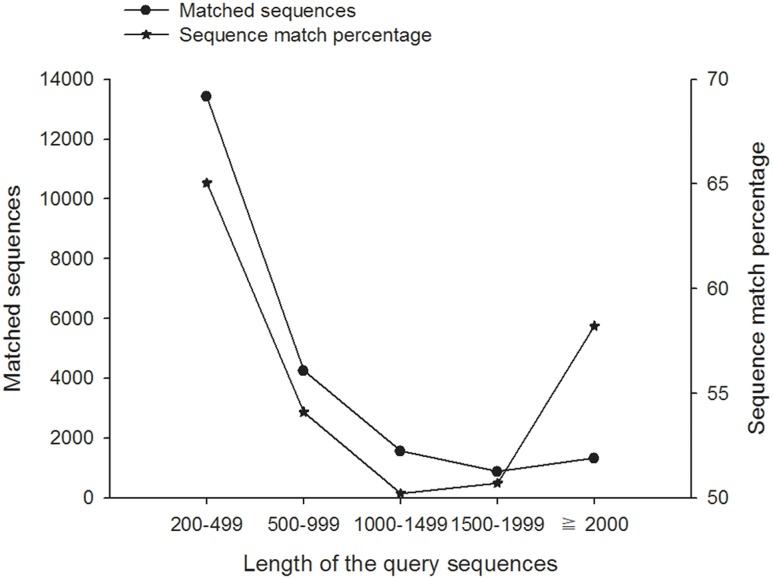
Effect of query unigene length on the numbers of matched unigenes and the percentage of unigenes matched in NCBI nr database.

Putative functions of the assembled unigenes’ were annotated by different databases. In the NCBI nr database, unigenes were searched using BLASTX with a cut-off E-value of 1.0e-5, and 21,463 unigenes (60.19% of all unigenes) could annotate in the nr database (Table 1). Figure 2 shows the effect of length of unigenes on matching unigenes and the percentage of unigenes matched on the NCBI nr database. The proportion of unigenes with matches in the nr database was between 50 to 65% in different ranges of unigene length. The lowest match percentage (50.22%) was unigenes between 1000 to 1499 bp. The unigene match percentage decreased with increasing length of unigenes when the length was less than 1000 bp, but, when the length of unigenes was longer than 1500 bp, the unigene match percentage increased with increasing length of unigenes. The highest match efficiency (65.04%) was obtained for unigenes between 200 to 499 bp (Figure 2). The unigene match percentage pattern in this study is a little different than in other studies. Usually, unigene match percentage of the nr database increases with the size of the assembled sequences, and the percentage is usually more than 80% when the size is longer than 1500 bp [6], [21], [34]. This difference may be cause by different samples, because we used antennae, but, in other studies, plants or the whole body of insects were used. Moreover, some of longer unigenes that had no BLAST hits might represent potential antenna-specific genes.

**Table 1:** Numbers of unigenes annotating to public databases.

NR	NT	Swiss-port	KEGG	COG	GO	ALL
21,463	9,526	16,350	14,499	6,625	11,154	22,134

**Figure 3:**
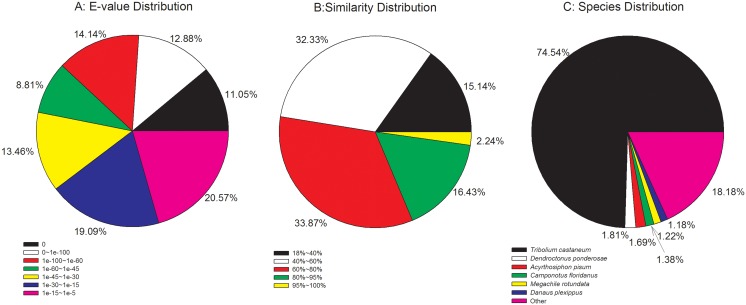
Characteristics of homology search of assembled unigenes against the nr database. (A) E-value distribution; (B) Similarity distribution; (C) Species distribution; the first hits of each unigene were used for analysis.

**Figure 4:**
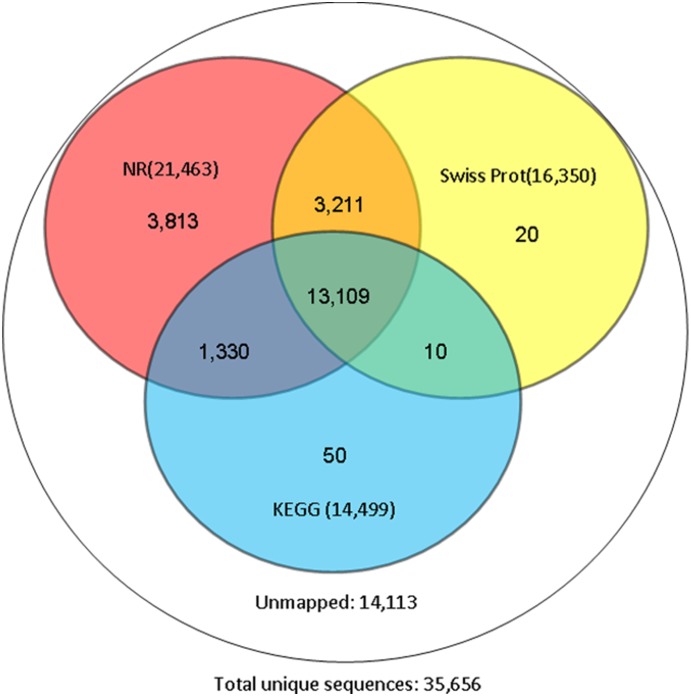
Unigene hits against the three main public databases.

Figure 3 shows the characteristics of unigenes against the nr database. We found that 46.88% of the mapped unigenes have strong homology (the top hits: E-value smaller than 1.0E-45), whereas 53.12% of the unigenes ranged between 1.0E-5 to 1.0E-45 (Figure 3A). For similarity distribution, the percentage of similarity higher than 80% was 18.67%, while similarity ranging from 60% to 80% was 33.87% and similarity ranging from 18% to 60% was 47.47% (Figure 3B). The species distribution showed 74.54% of the unigenes have top matches (first hit) with sequences from the Coleoptera species *Tribolium castaneum*, followed by the Coleoptera species *Dendroctonus ponderosae* (1.81%), Hemiptera species *Acyrthosiphon pisum* (1.69%) and Hymenoptera species *Camponotus floridanus* (1.38%) (Figure 3C). The species distribution not only depends on the genetic differentiation between the submission and the species deposited in the nr database, but also the number of genes deposited of one species. In this study, almost three fourths of the unigenes had first matches with *T. castaneum* due to *A. corpulenta* and *T. castaneum* belonging to the same order Coleoptera, and *T. castaneum* is unique in this order in having genome information available. The annotation results showed that there are 16,350 and 14,499 unigenes that could be annotated to Swiss-Prot and KEGG databases, respectively (Table 1). In all compared databases, 22,134 out of 35,656 unigenes’ functions were annotated (Table 1). Figure 4 shows the results of unigenes against three main public databases, and 21,543 (60.42%) unigenes were annotated by those databases. Among the unigenes, 13,109 (36.77%) unigenes could be annotated by all three databases, 16,320 (45.77%) were annotated by NR and Swiss-Prot, 14,439 (40.50%) by NR and KEGG, and 13,119 (36.79%) by Swiss-Prot and KEGG; however, 14,113 (39.58%) unigenes could not annotate in these three databases (Figure 4).

### GO, COG classification and KEGG identification

**Figure 5:**
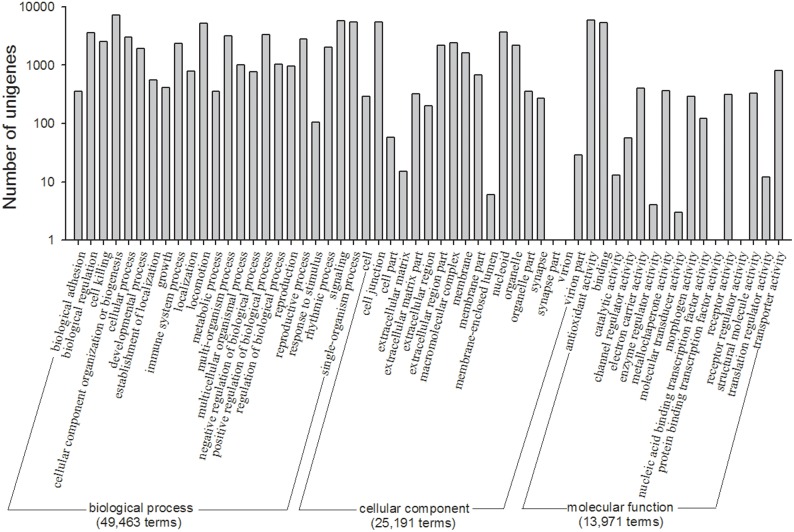
11,154 unigenes were hits in Gene Ontology (GO) classification. The results are assigned in three main categories: biological process, cellular component and molecular function. The x-axis indicates the sub-categories and the y-axis indicates the number of unigenes in that category.

The functions of the unigenes were classified by GO assignments. Based on sequence homology, 11,154 (31.28%) unigenes could be assigned 88,625 GO term annotations. Figure 5 shows that 49,463, 25,191 and 13,971 GO terms assigned to the three main categories (biological process, cellular component and molecular function), respectively. Three main categories were further divided into 57 functional groups, and the terms number of ‘cellular process’ (7,230 terms) was the largest for ‘biological process’, ‘cell’ and ‘cell part’ (both 5,447 terms) for ‘cellular component’, and ‘binding’ (5859 terms) for ‘molecular function’; otherwise, there was only one term in the clusters of ‘virion’, ‘virion part’, ‘protein tag’ and ‘receptor regulator activity’, three terms in ‘morphogen activity’, and four terms in ‘metallochaperone activity’ (Figure 5).

**Figure 6:**
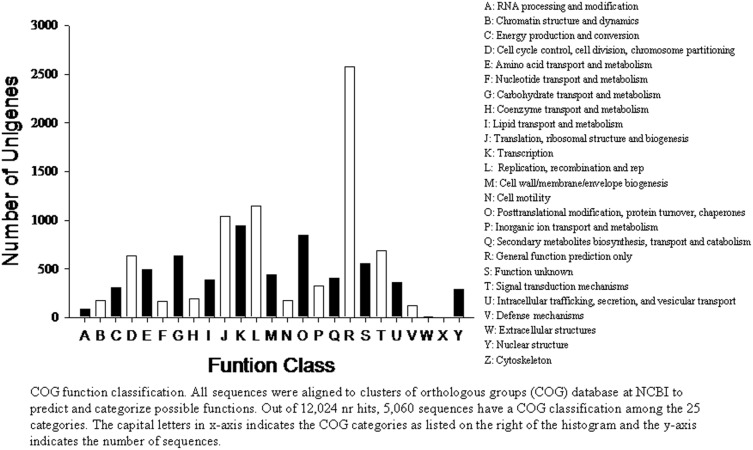
COG function classification. All unigenes were aligned to clusters of orthologous groups (COG) database at NCBI to predict possible functions. Out of 21,463 nr hits, 6,255 sequences had a COG classification among the 25 categories. The capital letters in x-axis indicate the COG categories as listed on the right and the y-axis indicates the number of unigenes in each category.

The nr annotated unigenes were further searched for the genes included in the different COG classifications. COG classification shows that 6,625 out of 21,463 nr hits could be categorized (Table 1). In the 25 COG categories, the largest group was the cluster for ‘general function prediction only’ (2,575, or 38.87%) followed by ‘replication, recombination and repair’ (1142, 17.24%) ‘translation, ribosomal structure and biogenesis’ (1044, 15.76%) and ‘transcription’ (941, 14.20%), while the categories of ‘nuclear structure’ (4, 0.06%), ‘extracellular structures’ (10, 0.16%) and ‘RNA processing and modification’ (87, 1.39%) were the smallest clusters (Figure 6).

To identify the active biological pathways in *A. corpulenta*, unigenes were mapped to the reference pathways in the KEGG [35]. As a result, 258 KEGG pathways were identified and 14,499 (40.66% of 35,656) unigenes were assigned to different KEGG pathways. The most assigned pathways by the unigenes were ‘metabolic pathways’ (1955, 13.48%), ‘pathways in cancer’ (607, 4.19%) and ‘purine metabolism’ (526, 3.63%). The KEGG pathway provides basic information when determining the specific processes and pathways during *A. corpulenta* research. For instance, there were 62 unigenes that were sorted into olfactory transduction, and this pathway is closely related to insect chemodetection.

### Expression level of unigenes

**Figure 7:**
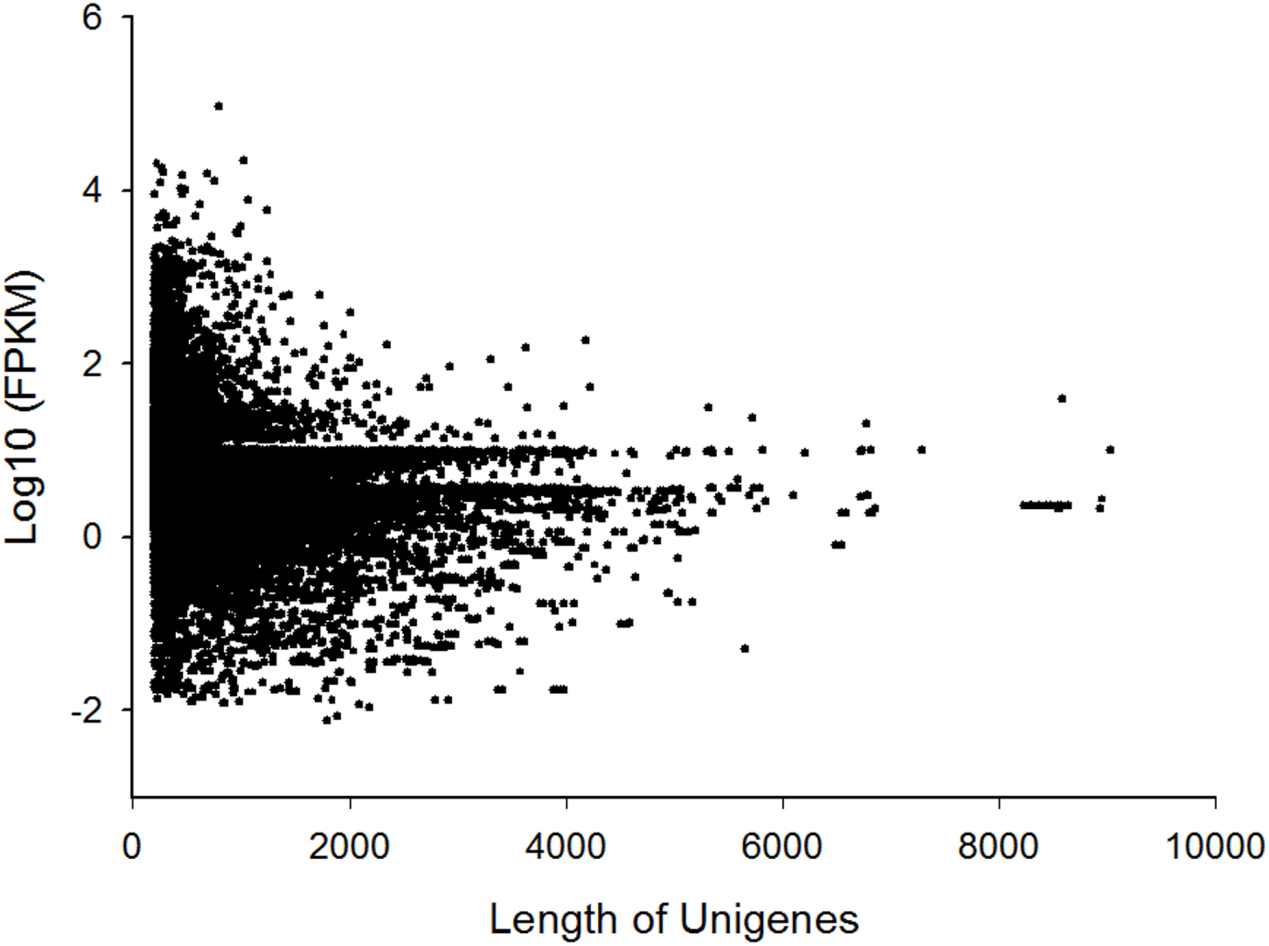
FPKM values versus different lengths of unigenes.

**Figure 8:**
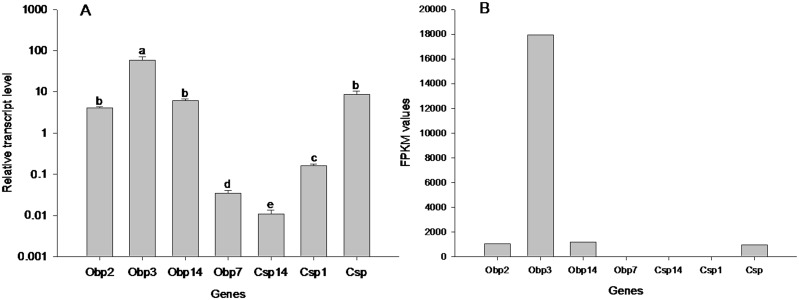
Relative expression of OBPs and CSPs genes to GAPDH in *Anomala corpulenta* (A) and the FPKM values of those genes (B). Bars denoted by the same letter indicate that there are no significant differences in gene expression in antenna (Tukey’s b P>0.05).

FPKM value was used to estimate the unigene expression levels [30]. The results showed 3,149 (8.83%) in 35,656 unigenes had FPKM values less than 1, 21,077 (59.11%) had FPKM values between 1 and 10, and 10,636 (29.83%) had FPKM values between 10 and 100. The FPKM values of 794 unigenes (2.23%) were greater than 100, and even 11 unigenes had FPKM values larger than 10,000 (Figure 7). In the top most expressed unigenes according to FPKM value (Table S3 in Data S1), one of them annotated as pheromone binding protein and another annotated as odorant binding protein 3, which is reasonable given that this is an antennal transcriptome. Besides the chemodetection protein, there were 2 aerobic metabolism-related genes (unigene 22007 and 15220 annotated as cytochrome C oxydase subunit I and cytochrome C oxidase subunit III, respectively), and this indicated that the antennae is a very active tissue. The amplification efficiencies of each pair of primers is shown in Figure S1 in Data S1. Figure 8 shows the expression level calculated by RT-qPCR and FPKM for 4 OBPs and 3 CSPs genes. The RT-qPCR results were agreement with the results of FPKM (Figure 8). RT-qPCR shows the expression level of OBP3 (unigene17160) is the highest followed by CSP (unigene14842), and the expression levels of OBP7 (unigene10159), CSP14 (unigene6242) and CSP1 (unigene6244) were very low.

### Chemodetection related unigenes

**Figure 9:**
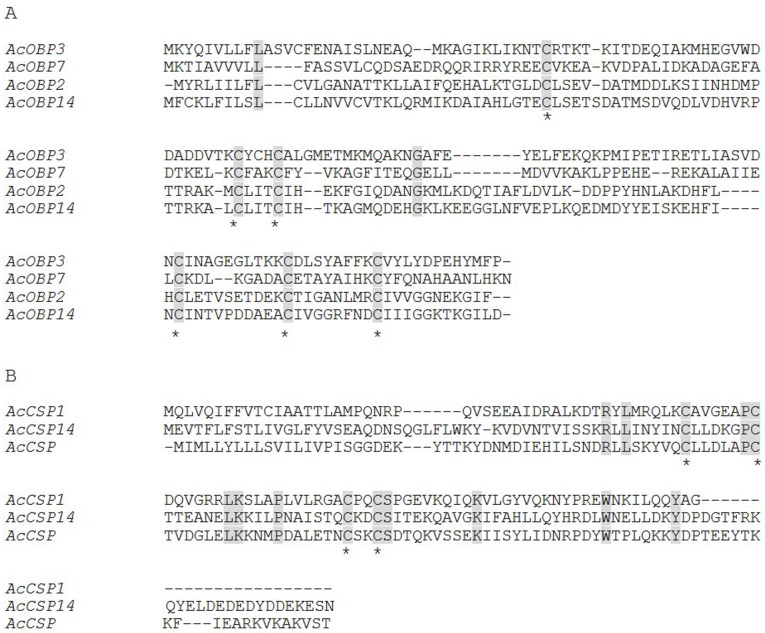
Alignment of amino acid sequences of OBPs (A) and CSPs (B) from *A. corpulenta*. Conserved residues were highlighted in gray and conserved cysteines were marked with “*” below the alignment.

Considering that chemodetection plays a key role in insect behavior, and determination of the specific genes involved in the pathway of odorant detection could be beneficial for control of *A. corpulenta* adults, we analyzed the unigenes related to chemodetection. The unigenes related to ORs (odorant receptors, olfactory receptors and chemosensory receptors), OBPs (odorant binding receptors), PBPs (pheromone binding proteins), CSPs (chemosensory proteins), PDEs (pheromone degrading enzymes), SNMPs (sensory neuron membrane proteins) and SAPs (sensory appendage proteins) were identified (Table 2). There were 93 related unigenes obtained for ORs, 24 for OBPs, 7 for PBPs and 6 for CSPs, and, in total, 167 unigenes’ functions were related to chemodetection. In this study, we detected 24 OBPs unigenes and 6 CSPs unigenes. *Nilaparvata lugens* antenna transcriptome contained 10 OBPs and 11 CSPs [36], while for the *T. castaneum* genome which is the only known genomic sequence in the Coleoptera, has 19 CSPs and 19 OBPs. The OBPs we obtained from *A. corpulenta* antenna is a little more than in *T. castaneum*, but the number of CSPs is much less than in *T. castaneum*. This may indicate the CSPs have more functions beside chemodetection and exist in tissues other than the antenna. According to the unigenes’ sequences, we identified 4 OBPs and 3 CSPs’ full length cDNAs sequence (Table 3). The nucleotide sequences and deduced amino acid sequences are shown in Figure S2 in Data S1, and some traits of nucleotide and putative amino acid sequences of OBPs and CSPs are shown in Table S4 in Data S1. Alignment of OBPs and CSPs are shown in figure 9, and the alignment results show all OBPs from *A. corpulenta* have six cysteine residues conserved [37] and all CSPs have four cysteine residues conserved [38]. In OBPs, the function of six cysteine residues were considered to constitute three disulfide bridges, to further protect and bind small hydrophobic ligands [39], [40].

**Table 2:** Unigenes related to chemodetection.

Gene name	Number of unigenes with a hit in nr database
ORs (odor receptors)	93
OBPs (odorant binding receptors)	24
PBPs (pheromone binding proteins)	7
CSPs (chemosensory proteins)	6
PDEs (pheromone degrading enzymes)	31
SNMPs (sensory neuron membrane proteins)	2
SAPs (sensory appendage proteins)	4

Number of sequences obtained in this study with hits to the corresponding proteins in the NCBI nr database.

**Table 3:** Identified and sequence confirmed OBP and CSP unigenes.

Gene ID	OBPs or CSPs	Length (bp)	Subject ID	Species	E value
Unigene6275	OBP	618	BAC07271.1	*Heptophylla picea*	4e-14
Unigene17160	OBP	723	ADX96030.1	*Holotrichia oblita*	5e-58
Unigene17481	OBP	792	XP_008191538.1	*T. castaneum*	4e-12
Unigene10159	OBP	531	XP_975684.1	*T. castaneum*	2e-45
Unigene6242	CSP	549	NP_001039287.1	*T. castaneum*	5w-34
Unigene6244	CSP	745	NP_001039273.1	*T. castaneum*	1e-40
Unigene14842	CSP	584	AFI45003.1	*Dendroctonus ponderosae*	8–39

**Figure 10:**
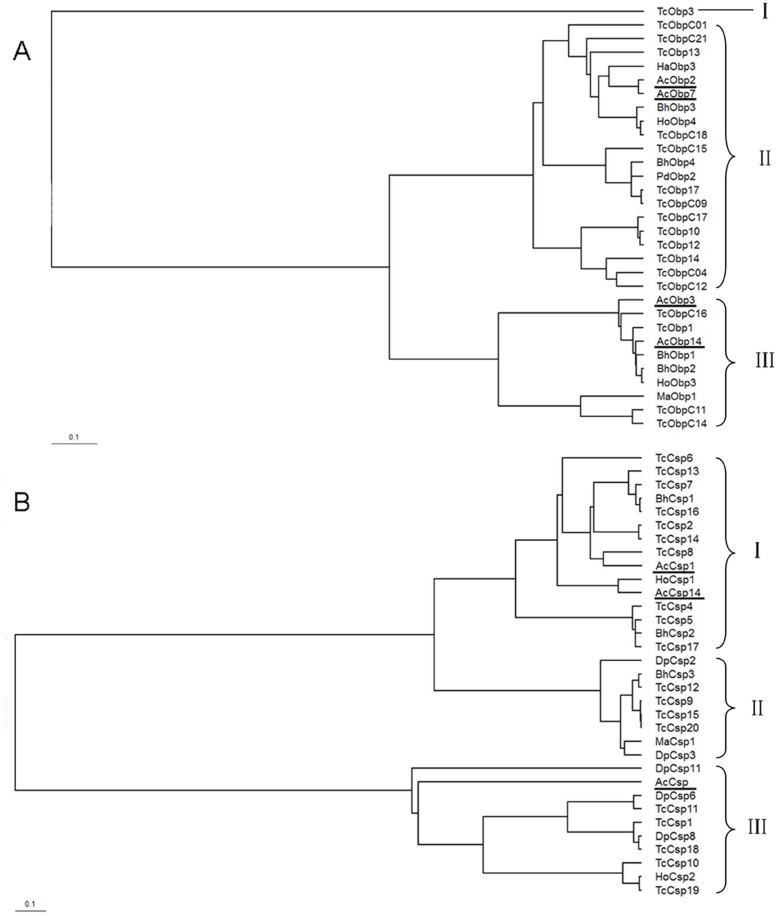
Phylogenetic tree of OBPs (A) and CSPs (B) from *Anomala corpulenta* and other Coleoptera species constructed by BEAST 2. The Obps and Csps from *A. corpulenta* were underlined. Bh: *Batocera horsfieldi*; Ha: *Harmonia axyridis*; Ho: *Holotrichia oblita*; Ma: *Monochamus alternatus*; Pd: *Phyllopertha diversa*; Tc: *Tribolium castaneum*; Dp: *Dendroctonus ponderosae*.

The phylogenetic tree constructed by BEAST 2 showed that both OBPs and CSPs in Coleoptera clustered into three groups, four OBPs and three CSPs from *A. corpulenta* were distributed into two of three groups (Figure 10). The OBPs and CSPs from *T. castaneum*, which was the only species with known genomic sequence from Coleoptera, were distributed into all three groups (Figure 10A, B). AcOBP2 and AcOBP7 showed high conservation and presumed orthology with a subset in group of OBPs (Figure 10A). The most conserved orthology subset for OBPs and CSPs wasn’t always from the same species (HoObp4 and TcObpC18, BhObp2 and HoObp3, and most Csps in Figure 10B). Phylogenetic analyses also suggested a *T. castaneum* specific clade which belongs to the OBPs group. Thus, most OBPs and CSPs (except AcObp2 and AcObp7) in *A. corpulenta* appear to be paralogous with other species, and indicated OBPs and CSPs may have evolved independently from their ancestors or these OBPs and CSPs may have diverged at an early time and/or are still evolving. Also, OBPs and CSPs in *A. corpulenta* being paralogous with other species may indicated that the different clades have different functions, and it will be interesting to determine whether the various members of the OBPs and CSPs belonging to different clades display different functional, as well as structural, homology.

## Conclusion

In the present study, we used Illumina sequencing technology to sequence the antennal transcriptome. The single run produced more than 35,656 unigenes with 21,463 unigenes having an above cut-off BLAST result. 4 OBPs and 3 CSPs’ unigenes were identified and confirmed by RT-PCR and Sanger sequencing technology, which shows that next generation sequencing technology could be a reliable technology to develop genomic resources that are currently unavailable. The results of OBPs and CSPs RT-qPCR agreeing with the FPKM values of those unigenes proves that the FPKM value could be a good tool to predict the expression of unigenes. These findings provide a substantial contribution to existing sequence resources for *A. corpulenta*. The sequences related to odorant detection and their functional categorization could provide a substantial foundation for research on *A. corpulenta*.

## Supporting Information

Data S1 Contains the following files: Figure S1. The Real-time PCR amplification efficiencies of one cycle were calculated according to the equation: E = 5^[-1/slope]^. All pair of primers have a high linearity (R2>0.985). Figure S2. Nucleotide sequences and deduced amino acid sequences for OBPs and CSPs. Comfirming primers were highlight in red and primers for RT-qPCR were in blue. Table S1. The primers to comfirm unigenes and RT-qPCR. Table S2. Summary of the Anomala corpulenta Transcriptome. Table S3. 11 most expressed unigenes according to FPKM value. Table S4. Traits of nucleotide and putative amino acid sequences of OBPs and CSPs.
(ZIP)
