# Correction: *De novo* Sequencing, Assembly and Characterization of Antennal Transcriptome of *Anomala corpulenta* Motschulsky (Coleoptera: Rutelidae)

**DOI:** 10.1371/journal.pone.0127303

**Published:** 2015-04-30

**Authors:** Haoliang Chen, Lulu Lin, Minghui Xie, Guangling Zhang, Weihua Su

Fig 6 and its legend are incorrect. Please view the correct Fig 6 and its legend here.

**Fig 6:**
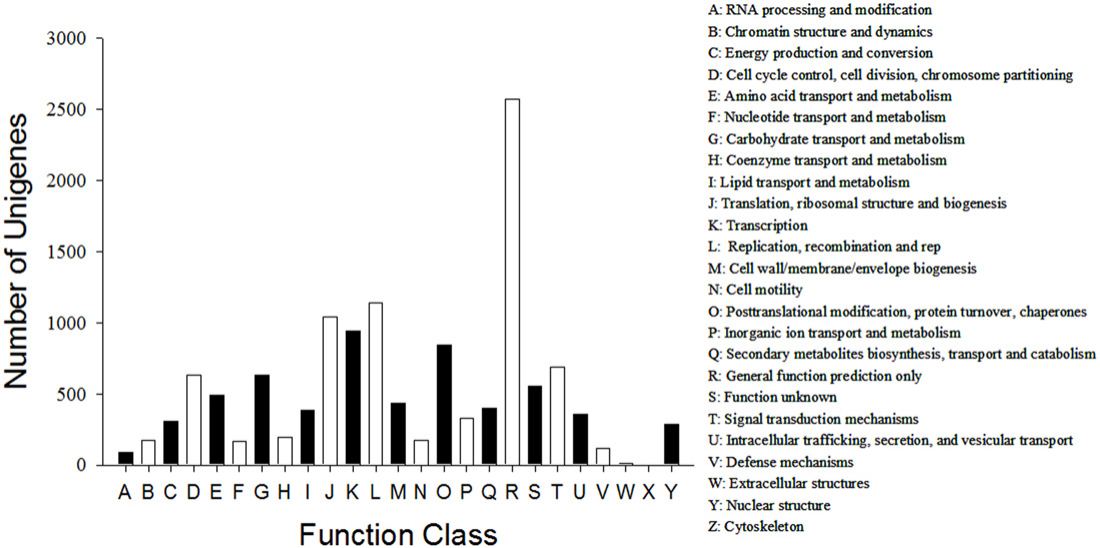
COG function classification. All unigenes were aligned to clusters of orthologous groups (COG) database at NCBI to predict possible functions. Out of 21,463 nr hits, 6,625 sequences had a COG classification among the 25 categories. The capital letters in x-axis indicate the COG categories as listed on the right and the y-axis indicates the number of unigenes in each category.
