# Supplementary material for: De novo Sequencing, Assembly and Characterization of Antennal Transcriptome of Anomala corpulenta Motschulsky (Coleoptera: Rutelidae)
Source: PLoS One. 2014 Dec 2;9(12):e114238. doi: 10.1371/journal.pone.0114238 (PMC4252136; doi:10.1371/journal.pone.0114238)
Supplement: Data S1 — Contains the following files: Figure S1. The Real-time PCR amplification efficiencies of one cycle were calculated according to the equation: E = 5[-1/slope]. All pair of primers have a high linearity (R2>0.985). Figure S2. Nucleotide sequences and deduced amino acid sequences for OBPs and CSPs. Comfirming primers were highlight in red and primers for RT-qPCR were in blue. Table S1. The primers to comfirm unigenes and RT-qPCR. Table S2. Summary of the Anomala corpulenta Transcriptome. Table S3. 11 most expressed unigenes according to FPKM value. Table S4. Traits of nucleotide and putative amino acid sequences of OBPs and CSPs. (ZIP) [file pone.0114238.s001.zip › Supporting Information/Figure S2.doc]

```
odorant binding protein 2(Unigene6275)
CAAAAAAATACTACTTGAAAGCGTATATAAAGAAGTAAACACTTTTTGACATTAGATCAGTATAATACAAA   71
ATGTACCGCTTGATTATTTTATTTCTTTGTGTTTTGGGAGCCAATGCTACAACGAAGTTGCTAGCAATATTTCAAGAGCATGCTCTGAAGACTGGTTTGGAC  173
M  Y  R  L  I  I  L  F  L  C  V  L  G  A  N  A  T  T  K  L  L  A  I  F  Q  E  H  A  L  K  T  G  L  D     34
TGCCTCTCTGAAGTCGATGCTACAATGGATGATCTAAAATCAATCATAAACCACGATATGCCAACAACTCGTGCGAAAATGTGCTTAATAACGTGTATACAT  275
C  L  S  E  V  D  A  T  M  D  D  L  K  S  I  I  N  H  D  M  P  T  T  R  A  K  M  C  L  I  T  C  I  H     68
GAAAAATTTGGAATCCAAGATGCCAACGGTAAAATGTTGAAAGATCAAACTATAGCCTTTCTTGATGTACTTAAAGATGATCCACCATATCACAATCTCGCT  377
E  K  F  G  I  Q  D  A  N  G  K  M  L  K  D  Q  T  I  A  F  L  D  V  L  K  D  D  P  P  Y  H  N  L  A    102
AAGGATCATTTTCTACACTGCTTAGAAACAGTATCTGAAACTGACGAGAAGTGTACGATTGGAGCTAACCTCATGCGCTGTATAGTTGTTGGAGGAAACGAA  479
K  D  H  F  L  H  C  L  E  T  V  S  E  T  D  E  K  C  T  I  G  A  N  L  M  R  C  I  V  V  G  G  N  E    136
AAAGGCATTTTTTAATGTTTAAAAAATCTGCTAGTTACAATTCAATAGTATTGTATTGCTATCAATTTATGTATATGTATTAGTGCTTCAAAACAAAAAAAA  581
K  G  I  F                                                                                                                140
AAAAAAAAAAAAAAAAAAA                                                                                                      600

odorant binding protein 3(Unigene 17160)
                                                                                            GTTGAAACTATACATTCCCAATAAT  25
TATTCATCGTAATTGATACCGTCCGATGAATAGACATGTATAAAAGAAATCACAAAATCGTGCAGTCAATTTTGATAACGAATCAATCATCACGTAATCATA 127
ATGAAGTATCAAATTGTCTTATTGTTTTTAGCGAGTGTGTGCTTTGAAAATGCAATTTCGTTGAATGAAGCACAAATGAAAGCTGGCATAAAATTAATTAAA  229
M  K  Y  Q  I  V  L  L  F  L  A  S  V  C  F  E  N  A  I  S  L  N  E  A  Q  M  K  A  G  I  K  L  I  K     34
AATACATGCAGGACAAAAACTAAAATCACTGATGAGCAAATCGCAAAAATGCACGAAGGTGTTTGGGATGATGCTGATGACGTAACAAAGTGTTATTGTCAC  331
N  T  C  R  T  K  T  K  I  T  D  E  Q  I  A  K  M  H  E  G  V  W  D  D  A  D  D  V  T  K  C  Y  C  H     68
TGCGCTTTGGGTATGATGAAAATGCAAGCTAAGAACGGAGCTTTTGAATATGAACTTTTCGAAAAACAGAAACCGATGATACCGGAAACGATAAGGGAGACT  433
C  A  L  G  M  M  K  M  Q  A  K  N  G  A  F  E  Y  E  L  F  E  K  Q  K  P  M  I  P  E  T  I  R  E  T    102
TTAATAGCAAGTGTAGATAATTGCATCAATGCGGGAGAAGGGTTGACGAAAAAATGTGACCTATCTTATGCCTTCTTCAAATGCGTTTACTTATACGATCCT  535
L  I  A  S  V  D  N  C  I  N  A  G  E  G  L  T  K  K  C  D  L  S  Y  A  F  F  K  C  V  Y  L  Y  D  P    136
GAGCATTACATGTTCCCGTGATATAGTCATAACTACCGAACAATTTTCCGTAGTTTAATTCTAATTTGTATGTAGTTTAATATCACTGATGTAATGAGTATA  637
E  H  Y  M  F  P                                                                                                         142
TATGTATATGTTCTAGTTAATGTATTTACTAATGTAAAAATAATAAATCTTTTGTTTCTGTCCAAAAAAAAAAAAAAAAAAAAAAA                     723

odorant binding protein 14(Unigene 17481)

AAGATGTTTTACTTCATAATATTAAACCAAATAAAGTTACACGATTTTGGAAGAACC  57
ATGTTCTGTAAACTATTTATACTAAGCCTGTGTTTGCTCAATGTGGTATGTGTAACAAAGCTTCAGCGAATGATAAAAGATGCTATTGCTCACCTTGGAACT  159
M  F  C  K  L  F  I  L  S  L  C  L  L  N  V  V  C  V  T  K  L  Q  R  M  I  K  D  A  I  A  H  L  G  T     34
GAATGCTTATCAGAAACTTCTGATGCTACCATGTCGGACGTACAAGACTTGGTAGATCATGTAAGACCAACGACACGTAAAGCACTGTGTTTAATAACATGT  261
E  C  L  S  E  T  S  D  A  T  M  S  D  V  Q  D  L  V  D  H  V  R  P  T  T  R  K  A  L  C  L  I  T  C     68
ATACACACAAAAGCTGGAATGCAAGATGAACATGGGAAATTAAAAGAAGAAGGTGGTTTAAATTTCGTGGAACCACTTAAGCAAGAAGATATGGACTATTAC  363
I  H  T  K  A  G  M  Q  D  E  H  G  K  L  K  E  E  G  G  L  N  F  V  E  P  L  K  Q  E  D  M  D  Y  Y    102
GAAATATCAAAAGAACATTTTATAAATTGTATAAATACCGTTCCCGATGATGCTGAAGCATGTATTGTTGGTGGTCGATTTAACGATTGTATTATAATTGGA  465
E  I  S  K  E  H  F  I  N  C  I  N  T  V  P  D  D  A  E  A  C  I  V  G  G  R  F  N  D  C  I  I  I  G    136
GGAAAAACAAAAGGTATATTAGATTAAAAACTTTTGCTTCAATCTACCGTAATTTTAGATTAATTGTATGATGTATGGTGGAAAGTGGTACAAAAATGTAAA  567
G  K  T  K  G  I  L  D                                                                                                  144
ATACTTTTAAAAAGTAATTTTGTTTATAATGGAGCATTAACATGTACTTATAGGTTTTATTAACTACAACGATTATCCATAAAATACATATTTTTGAATCAA  669
ACGCAATTATTTTTAGTATTTAGTTTCAGTTTTAATAAAAAGTTGAATATATACTGAAAACTAAAAAAAGTATGCTATATTAAATAGAAATTACGACAAACG  771
AGATTTGATAAAATAAAAAAA                                                                                                   792


odorant binding protein 7(Unigene 10159)
AGTTGTTACCTGACAAGCCTAACAGA   26
ATGAAGACTATTGCGGTAGTCGTATTGTTATTCGCATCTTCAGTTCTGTGTCAAGATTCCGCAGAAGACAGGCAACAACGTATACGAAGATATCGCGAGGAA  128
M  K  T  I  A  V  V  V  L  L  F  A  S  S  V  L  C  Q  D  S  A  E  D  R  Q  Q  R  I  R  R  Y  R  E  E     34
TGCGTAAAGGAAGCCAAGGTTGATCCAGCACTCATAGATAAGGCGGATGCTGGCGAATTCGCAGATACTAAGGAACTGAAGTGTTTTGCCAAATGTTTCTAC  230
C  V  K  E  A  K  V  D  P  A  L  I  D  K  A  D  A  G  E  F  A  D  T  K  E  L  K  C  F  A  K  C  F  Y     68
GTGAAAGCCGGTTTTATCACTGAACAAGGCGAACTACTCATGGATGTTGTGAAAGCAAAACTTCCTCCGGAACATGAACGTGAAAAGGCTTTGGCGATTATT  332
V  K  A  G  F  I  T  E  Q  G  E  L  L  M  D  V  V  K  A  K  L  P  P  E  H  E  R  E  K  A  L  A  I  I    102
GAACTGTGCAAGGACCTGAAAGGAGCGGATGCCTGCGAAACAGCGTACGCCATTCACAAATGTTATTTCCAGAACGCCCACGCAGCTAACTTGCACAAAAAT  434
E  L  C  K  D  L  K  G  A  D  A  C  E  T  A  Y  A  I  H  K  C  Y  F  Q  N  A  H  A  A  N  L  H  K  N    136
TAAAATATAAAAATGTGACCTGTATTATCAGTATGTAAAAATTACTAACATTACAAGAACGAGTATAGCTTTAAAACTGTTCCATTAAATGAAAATT        531
```

```
chemosensory protein 14(Unigene 6242)
GTGATACCCGTCTGCCTTGAAAAGCATACCAAATGAAAACAAAAATATTTTAAACAAAATTAGTTCGAGATACGTTAATCAAA   83
ATGGAAGTAACATTTCTGTTTTCTACTCTTATCGTCGGTTTATTTTACGTGAGTGAAGCTCAAGATAACTCACAGGGCCTTTTTCTATGGAAATACAAGGTG  185
M  E  V  T  F  L  F  S  T  L  I  V  G  L  F  Y  V  S  E  A  Q  D  N  S  Q  G  L  F  L  W  K  Y  K  V     34
GACGTCAATACTGTAATATCCAGCAAACGTTTATTAATAAATTACATCAATTGCTTATTAGATAAAGGGCCATGTACTACTGAAGCTAACGAACTTAAAAAA  287
D  V  N  T  V  I  S  S  K  R  L  L  I  N  Y  I  N  C  L  L  D  K  G  P  C  T  T  E  A  N  E  L  K  K     68
ATACTACCTAATGCCATATCGACTCAGTGTAAGGATTGCAGTATAACGGAGAAACAAGCTGTCGGAAAAATATTCGCGCACTTATTACAATACCACCGAGAT  389
I  L  P  N  A  I  S  T  Q  C  K  D  C  S  I  T  E  K  Q  A  V  G  K  I  F  A  H  L  L  Q  Y  H  R  D    102
CTATGGAATGAATTATTAGATAAATACGATCCCGATGGAACATTTAGAAAACAATACGAATTAGACGAAGATGAAGATTACGACGACGAAAAAGAATCTAAT  491
L  W  N  E  L  L  D  K  Y  D  P  D  G  T  F  R  K  Q  Y  E  L  D  E  D  E  D  Y  D  D  E  K  E  S  N    136
TGAAAGGGTATTCATAAAATATTGTAGTTTATGTATTTTCAAATAAATTTTATTAATC                                                       549


chemosensory protein 1(Unigene 6244)
TACGACTCCAGGAGGTTGGCGAGATAAGGTCC  32
CCTGCCTGACTGTTATATAGCGAGGCGCATTTTTAAACATTAGACGGTCTCAGGTAGTCCGGTCGAAACGGTGCCACTATTATAATATAGCGCCAAAGCAAA  134
AGGCTTCAGTTTAACGAGGTCGATTCTCTATATCAGTGCTACCAACATCACTTTCTCCATCACTTTCTTCCTGAACATCAGCTAGGTAACATCAACATAACC  236
ATGCAACTAGTACAGATATTTTTCGTAACTTGTATCGCTGCGACAACGCTGGCCATGCCGCAGAATCGACCGCAGGTATCTGAAGAAGCTATCGATAGGGCC  338
M  Q  L  V  Q  I  F  F  V  T  C  I  A  A  T  T  L  A  M  P  Q  N  R  P  Q  V  S  E  E  A  I  D  R  A     34
CTCAAGGATACTAGATATTTGATGAGACAGTTGAAGTGCGCGGTCGGTGAAGCACCCTGTGATCAAGTTGGTCGCAGATTGAAAAGTCTAGCACCTTTAGTT  440
L  K  D  T  R  Y  L  M  R  Q  L  K  C  A  V  G  E  A  P  C  D  Q  V  G  R  R  L  K  S  L  A  P  L  V     68
CTGCGGGGGGCTTGTCCACAATGCAGTCCCGGTGAAGTTAAACAAATCCAGAAGGTCTTAGGTTACGTACAGAAGAATTATCCACGAGAATGGAATAAAATA  542
L  R  G  A  C  P  Q  C  S  P  G  E  V  K  Q  I  Q  K  V  L  G  Y  V  Q  K  N  Y  P  R  E  W  N  K  I    102
TTACAACAGTACGCCGGATAAAACGATCCAAAAACAAACAAACTAGTACAAAATTAATTTCCCAATGTCTTCTTTTTTTACTGTAATTATAATAGCTTAATT  644
L  Q  Q  Y  A  G                                                                                                         108
ATAGAGTTTGTAGGATGTAATTAGCTAATTGTAAGTAGACCACTTTTGTTTTGTGTAACTTGTAAATGTATTATTAGACCGTAATGTAATAATAAATCCTC   745

chemosensory protein(Unigene 14842)
CTCTTATATACACACATGCA   20
CATTAACTATCAAATTGTGGTTATCACAACATCGCTACGTAATCATTCAGTATTTTGCATATGTGTATATAAGAGAGTGTTATTGAGAACAGATTTATACGA  122
ATGATCATGTTACTCTACTTACTTTTGAGTGTTATCCTAATAGTTCCGATCTCTGGGGGCGACGAAAAGTATACTACAAAATATGATAATATGGATATAGAG  224
M  I  M  L  L  Y  L  L  L  S  V  I  L  I  V  P  I  S  G  G  D  E  K  Y  T  T  K  Y  D  N  M  D  I  E     34
CATATTTTATCCAATGATCGTTTACTGTCTAAATATGTTCAGTGTTTATTAGATTTGGCTCCTTGTACTGTGGATGGACTCGAACTAAAAAAAAACATGCCT  326
H  I  L  S  N  D  R  L  L  S  K  Y  V  Q  C  L  L  D  L  A  P  C  T  V  D  G  L  E  L  K  K  N  M  P     68
GATGCTTTAGAAACCAATTGTTCTAAATGTTCTGATACACAGAAGGTTAGTTCTGAAAAGATTATTTCTTATTTAATTGATAATAGACCAGATTATTGGACA  428
D  A  L  E  T  N  C  S  K  C  S  D  T  Q  K  V  S  S  E  K  I  I  S  Y  L  I  D  N  R  P  D  Y  W  T    102
CCATTACAAAAAAAATACGATCCTACTGAGGAATATACCAAGAAGTTTATAGAGGCTAGGAAGGTAAAGGCTAAAGTATCGACTTAATATGTAATTAATATA  530
P  L  Q  K  K  Y  D  P  T  E  E  Y  T  K  K  F  I  E  A  R  K  V  K  A  K  V  S  T                          130
GAGTGAACACGTAATTAAAATGTTATTTTTCGTGAAAAAAAAAAAAAAAAAAAA                                                            584


Figure S2 Nucleotide sequences and deduced amino acid sequences for OBPs and CSPs. Comfirming primers were highlight in red and primers for RT-qPCR were in blue.
```
