# Supplementary material for: De novo Sequencing, Assembly and Characterization of Antennal Transcriptome of Anomala corpulenta Motschulsky (Coleoptera: Rutelidae)
Source: PLoS One. 2014 Dec 2;9(12):e114238. doi: 10.1371/journal.pone.0114238 (PMC4252136; doi:10.1371/journal.pone.0114238)
Supplement: Data S1 — Contains the following files: Figure S1. The Real-time PCR amplification efficiencies of one cycle were calculated according to the equation: E = 5[-1/slope]. All pair of primers have a high linearity (R2>0.985). Figure S2. Nucleotide sequences and deduced amino acid sequences for OBPs and CSPs. Comfirming primers were highlight in red and primers for RT-qPCR were in blue. Table S1. The primers to comfirm unigenes and RT-qPCR. Table S2. Summary of the Anomala corpulenta Transcriptome. Table S3. 11 most expressed unigenes according to FPKM value. Table S4. Traits of nucleotide and putative amino acid sequences of OBPs and CSPs. (ZIP) [file pone.0114238.s001.zip › Supporting Information/Table S3.doc]

| Unigene number | FPKM value | Annotation function |
| --- | --- | --- |
| 22007 | 10142 | cytochrome C oxydase subunit I |
| 17429 | 10604 | ferritin 2 isoform 1 |
| 12700 | 12027 | hypothetical protein |
| 17402 | 12838 | No annotated |
| 23841 | 15050 | hypothetical protein PTSG |
| 475 | 15398 | Histidine-rich protein |
| 15220 | 15829 | cytochrome C oxidase subunit III |
| 17160 | 17917 | odorant binding protein 3 |
| CL889.Contig3 | 20061 | antennae-rich cytochrome P450 |
| 15103 | 21574 | pheromone binding protein |
| 23840 | 90830 | hypothetical protein |

Table S3 11 most expressed unigenes according to FPKM value
